# (2*E*)-3-[4-(Dimethyl­amino)­phen­yl]-1-(4-fluoro­phen­yl)prop-2-en-1-one

**DOI:** 10.1107/S1600536811000377

**Published:** 2011-01-08

**Authors:** Jerry P. Jasinski, Ray J. Butcher, B. P. Siddaraju, B. Narayana, H. S. Yathirajan

**Affiliations:** a Department of Chemistry, Keene State College, 229 Main Street, Keene, NH 03435-2001, USA; b Department of Chemistry, Howard University, 525 College Street NW, Washington, DC 20059, USA; c Department of Studies in Chemistry, University of Mysore, Manasagangotri, Mysore 570 006, India; d Department of Studies in Chemistry, Mangalore University, Mangalagangotri 574 199, India

## Abstract

The mean planes of the two benzene rings in the title compound, C_17_H_16_FNO, are twisted slightly, making a dihedral angle of 7.8 (1)°. The prop-2-en-1-one group is also twisted slightly with a C—C—C—O torsion angle of −11.6 (3)°. In the crystal, weak inter­molecular C—H⋯O inter­actions link pairs of mol­ecules, forming centrosymmetric dimers.

## Related literature

Chalcones are precursors of all flavonoid-type natural products in biosynthesis, see: Marais *et al.* (2005 ▶). For their pharmacological activity, see: Di Carlo *et al.* (1999 ▶) and for their anti­malarial activity, see: Ram *et al.* (2000 ▶); Troeberg *et al.* (2000 ▶). For the synthesis and biological activity of some fluorinated chalcone derivatives, see: Nakamura *et al.* (2002 ▶). For a review of anti-infective and anti-inflammatory chalcones, see: Nowakowska (2007 ▶) and for recent advances in therapeutic chalcones, see: Ni *et al.* (2004 ▶). For related structures, see: Butcher *et al.* (2006 ▶, 2007*a* ▶,*b* ▶); Harrison *et al.* (2006 ▶); Jasinski *et al.* (2009 ▶); Jing (2009 ▶); Sarojini *et al.* (2007 ▶). For standard bond lengths, see: Allen *et al.* (1987 ▶). 
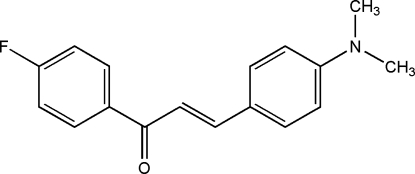


## Experimental

### 

#### Crystal data

C_17_H_16_FNO
*M* *_r_* = 269.31
Monoclinic, 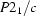

*a* = 12.8334 (3) Å
*b* = 12.3560 (2) Å
*c* = 9.3922 (2) Å
β = 105.965 (2)°
*V* = 1431.87 (5) Å^3^
*Z* = 4
Mo *K*α radiation
μ = 0.09 mm^−1^
*T* = 295 K
0.56 × 0.47 × 0.22 mm

#### Data collection

Oxford Diffraction Gemini R diffractometer
Absorption correction: multi-scan (*CrysAlis RED*; Oxford Diffraction, 2007 ▶) *T*_min_ = 0.675, *T*_max_ = 1.000
6644 measured reflections
2929 independent reflections
2098 reflections with *I* > 2σ(*I*)
*R*_int_ = 0.018

#### Refinement

*R*[*F* ^2^ > 2σ(*F* ^2^)] = 0.059
*wR*(*F* ^2^) = 0.197
*S* = 1.10
2929 reflections
184 parameters
H-atom parameters constrained
Δρ_max_ = 0.17 e Å^−3^
Δρ_min_ = −0.13 e Å^−3^

### 

Data collection: *CrysAlis PRO* (Oxford Diffraction, 2007 ▶); cell refinement: *CrysAlis PRO*; data reduction: *CrysAlis RED* (Oxford Diffraction, 2007 ▶); program(s) used to solve structure: *SHELXTL* (Sheldrick, 2008 ▶); program(s) used to refine structure: *SHELXTL*; molecular graphics: *SHELXTL*; software used to prepare material for publication: *SHELXTL*.

## Supplementary Material

Crystal structure: contains datablocks global, I. DOI: 10.1107/S1600536811000377/xu5139sup1.cif

Structure factors: contains datablocks I. DOI: 10.1107/S1600536811000377/xu5139Isup2.hkl

Additional supplementary materials:  crystallographic information; 3D view; checkCIF report

## Figures and Tables

**Table 1:** Hydrogen-bond geometry (Å, °)

*D*—H⋯*A*	*D*—H	H⋯*A*	*D*⋯*A*	*D*—H⋯*A*
C17—H17*A*⋯O1^i^	0.96	2.56	3.525 (3)	180

Symmetry code: (i) 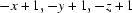.

**Fig. 1.**
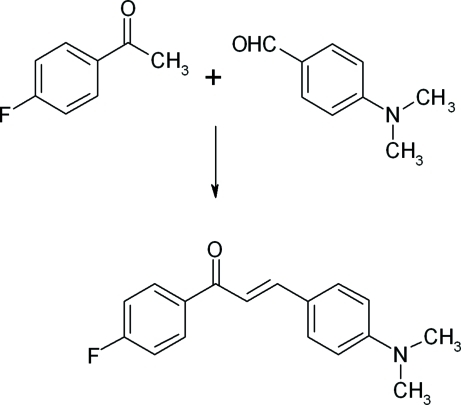
Reaction scheme for C17H16FNO.

**Fig. 2.**
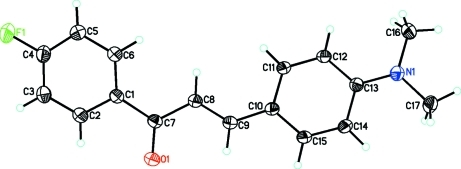
Molecular structure of the title compound showing the atom labeling scheme and 50% probability displacement ellipsoids.

**Fig. 3.**
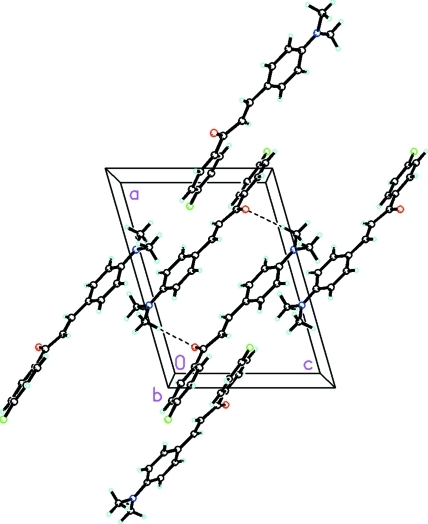
Packing diagram of the title compound viewed down the b axis. Dashed lines indicate weak a C—H···O intermolecular hydrogen bond interaction creating a layered structure along [101].

## supplementary crystallographic information

### Comment

Chalcones are known as the precursors of all flavonoid type natural products in biosynthesis (Marais *et al.*, 2005). Chalcones, one of the major classes of natural products with widespread distribution in fruits, vegetables, spices, tea and soy based foodstuff have been recently subjects of interest for their interesting pharmacological activities (Di Carlo *et al.*, 1999). Many chalcones have been described for their high antimalarial activity, probably as a result of michael addition of nucleophilic species to the double bond of the enone (Troeberg *et al.*, 2000 & Ram *et al.*, 2000). Synthesis and biological activities of some fluorinated chalcone derivatives is published (Nakamura *et al.*, 2002). A review of anti-infective and anti-inflammatory chalcones (Nowakowska, 2007) and recent advances in therapeutic chalcones have been reported (Ni *et al.*, 2004). The crystal structures of few related fluoro chalcones *viz*., 3-(3,4-dimethoxyphenyl)-1-(4-fluorophenyl)prop-2-en-1-one (Butcher *et al.*, 2006), (2E)-3-(4-fluorophenyl)-1-(3-hydroxyphenyl)prop-2-en-1-one (Butcher *et al.*, 2007*a*), (2E)-3-(4-fluorophenyl)-1-(4-methylphenyl)prop-2-en-1-one (Butcher *et al.*, 2007*b*), a second polymorph of (2E)-1-(4-fluorophenyl)-3-(3,4,5-trimethoxyphenyl)prop-2-en-1-one (Jasinski, *et al.*, 2009), (*E*)-3-(4-fluorophenyl)-1-phenyl-2-propen-1-one (Jing, 2009), 1-(4-fluorophenyl)-3-(4-methoxyphenyl)prop-2-en-1-one (Harrison *et al.*, 2006) and 3-(biphenyl-4-yl)-1-(4-fluorophenyl)prop-2-en-1-one (Sarojini *et al.*, 2007) have been reported. In a continuation of our studies and in view of the importance of fluoro chalcones, we report the synthesis and crystal structure of a new chalcone, C_17_H_16_FNO, (I).

The mean planes of the two benzene rings in the title compound, C_17_H_16_FNO, are twisted slightly being separated by 7.8 (0)° (Fig. 2). The prop-2-en-1-one group is also twisted slightly with a C2—C1—C7—O1 torsion angle of -11.6 (3)°. Bond distances and angles are in normal ranges (Allen *et al.*, 1987). A weak C—H···O intermolecular interaction (Table 1) contributes to crystal packing creating a centrosymmetric dimer (Fig. 3).

### Experimental

4-Fluoroaetophenone (1.38 g, 0.01 mol) was mixed with 4-(dimethylamino)benzaldehyde (1.49 g, 0.01 mol) and dissolved in ethanol (40 ml) (Fig. 1). To this solution 10 ml of KOH (30%) was added at 273 K. The reaction mixture stirred for 4 h and poured on to crushed ice. The resulting crude solid was filtered, washed successively with dilute HCl solution and distilled water and finally recrystallized from ethanol (95%) to give the pure chalcone. Crystals suitable for X-ray diffraction studies were grown by the slow evaporation of the solution of the compound in ethyl acetate (M.P.: 383–388 K). Composition: Found (Calculated) for C_17_H_16_FNO; C: 75.77 (75.82%); H: 5.96 (5.99%); N: 5.16 (5.20%).

### Refinement

All of the H atoms were placed in their calculated positions and then refined using the riding model with C—H = 0.93 Å (aromatic), or 0.96 Å (CH_3_). Isotropic displacement parameters for these atoms were set to 1.19–1.20 (aromatic) or 1.49 (CH_3_) times *U*_eq_ of the parent atom.

### Crystal data

**Table:**

C_17_H_16_FNO	*F*(000) = 568
*M**_r_* = 269.31	*D*_x_ = 1.249 Mg m^−^^3^
Monoclinic, *P*2_1_/*c*	Mo *K*α radiation, λ = 0.71073 Å
Hall symbol: -P 2ybc	Cell parameters from 3513 reflections
*a* = 12.8334 (3) Å	θ = 2.4–38.6°
*b* = 12.3560 (2) Å	µ = 0.09 mm^−^^1^
*c* = 9.3922 (2) Å	*T* = 295 K
β = 105.965 (2)°	Irregular triangular plate, yellow
*V* = 1431.87 (5) Å^3^	0.56 × 0.47 × 0.22 mm
*Z* = 4	

### Data collection

**Table:**

Oxford Diffraction Gemini R diffractometer	2929 independent reflections
Radiation source: fine-focus sealed tube	2098 reflections with *I* > 2σ(*I*)
graphite	*R*_int_ = 0.018
Detector resolution: 10.5081 pixels mm^-1^	θ_max_ = 26.7°, θ_min_ = 2.3°
φ and ω scans	*h* = −16→15
Absorption correction: multi-scan (*CrysAlis RED*; Oxford Diffraction, 2007)	*k* = −15→15
*T*_min_ = 0.675, *T*_max_ = 1.000	*l* = −11→11
6644 measured reflections	

### Refinement

**Table:**

Refinement on *F*^2^	Primary atom site location: structure-invariant direct methods
Least-squares matrix: full	Secondary atom site location: difference Fourier map
*R*[*F*^2^ > 2σ(*F*^2^)] = 0.059	Hydrogen site location: inferred from neighbouring sites
*wR*(*F*^2^) = 0.197	H-atom parameters constrained
*S* = 1.10	*w* = 1/[σ^2^(*F*_o_^2^) + (0.1067*P*)^2^ + 0.0852*P*] where *P* = (*F*_o_^2^ + 2*F*_c_^2^)/3
2929 reflections	(Δ/σ)_max_ < 0.001
184 parameters	Δρ_max_ = 0.17 e Å^−^^3^
0 restraints	Δρ_min_ = −0.13 e Å^−^^3^

### Special details

**Table:**

Geometry. All e.s.d.'s (except the e.s.d. in the dihedral angle between two l.s. planes) are estimated using the full covariance matrix. The cell e.s.d.'s are taken into account individually in the estimation of e.s.d.'s in distances, angles and torsion angles; correlations between e.s.d.'s in cell parameters are only used when they are defined by crystal symmetry. An approximate (isotropic) treatment of cell e.s.d.'s is used for estimating e.s.d.'s involving l.s. planes.
Refinement. Refinement of *F*^2^ against ALL reflections. The weighted *R*-factor *wR* and goodness of fit *S* are based on *F*^2^, conventional *R*-factors *R* are based on *F*, with *F* set to zero for negative *F*^2^. The threshold expression of *F*^2^ > σ(*F*^2^) is used only for calculating *R*-factors(gt) *etc*. and is not relevant to the choice of reflections for refinement. *R*-factors based on *F*^2^ are statistically about twice as large as those based on *F*, and *R*- factors based on ALL data will be even larger.

### Fractional atomic coordinates and isotropic or equivalent isotropic displacement parameters (Å^2^)

**Table:**

	*x*	*y*	*z*	*U*_iso_*/*U*_eq_	
F1	1.15377 (18)	0.10045 (18)	1.0627 (2)	0.1881 (10)	
O1	0.83748 (14)	0.47322 (14)	0.78907 (19)	0.1242 (6)	
C1	0.92349 (14)	0.30455 (16)	0.8178 (2)	0.0850 (5)	
C2	1.0044 (2)	0.3397 (2)	0.9375 (3)	0.1251 (9)	
H2A	1.0064	0.4124	0.9635	0.150*	
C3	1.0820 (3)	0.2720 (3)	1.0198 (4)	0.1402 (11)	
H3A	1.1363	0.2978	1.0999	0.168*	
C4	1.0777 (2)	0.1681 (3)	0.9820 (3)	0.1281 (9)	
C5	0.9972 (3)	0.1257 (3)	0.8685 (4)	0.1464 (12)	
H5A	0.9942	0.0519	0.8480	0.176*	
C6	0.9209 (2)	0.1953 (2)	0.7859 (3)	0.1187 (8)	
H6A	0.8665	0.1684	0.7068	0.142*	
N1	0.36368 (14)	0.37392 (15)	−0.0182 (2)	0.0984 (5)	
C7	0.84180 (15)	0.38300 (17)	0.7354 (2)	0.0894 (5)	
C8	0.76857 (15)	0.35318 (16)	0.5923 (2)	0.0853 (5)	
H8A	0.7756	0.2857	0.5520	0.102*	
C9	0.69177 (16)	0.42082 (15)	0.5183 (2)	0.0861 (5)	
H9A	0.6893	0.4870	0.5645	0.103*	
C10	0.61231 (15)	0.40735 (14)	0.3784 (2)	0.0810 (5)	
C11	0.60600 (15)	0.31682 (14)	0.2863 (2)	0.0831 (5)	
H11A	0.6573	0.2621	0.3149	0.100*	
C12	0.52677 (16)	0.30671 (15)	0.1558 (2)	0.0856 (5)	
H12A	0.5265	0.2460	0.0972	0.103*	
C13	0.44563 (15)	0.38556 (15)	0.1076 (2)	0.0828 (5)	
C14	0.45373 (18)	0.47770 (16)	0.1973 (2)	0.0968 (6)	
H14A	0.4037	0.5334	0.1684	0.116*	
C15	0.53447 (19)	0.48642 (15)	0.3267 (2)	0.0963 (6)	
H15A	0.5373	0.5488	0.3831	0.116*	
C16	0.3540 (2)	0.2790 (2)	−0.1099 (3)	0.1289 (9)	
H16A	0.4204	0.2681	−0.1364	0.193*	
H16B	0.2955	0.2886	−0.1980	0.193*	
H16C	0.3398	0.2171	−0.0563	0.193*	
C17	0.2862 (2)	0.4610 (2)	−0.0696 (3)	0.1149 (7)	
H17A	0.2522	0.4790	0.0065	0.172*	
H17B	0.2321	0.4381	−0.1569	0.172*	
H17C	0.3233	0.5234	−0.0922	0.172*	

### Atomic displacement parameters (Å^2^)

**Table:**

	*U*^11^	*U*^22^	*U*^33^	*U*^12^	*U*^13^	*U*^23^
F1	0.1983 (18)	0.1869 (19)	0.1399 (14)	0.0835 (16)	−0.0196 (14)	0.0050 (13)
O1	0.1291 (12)	0.1024 (11)	0.1227 (12)	0.0113 (9)	0.0033 (10)	−0.0346 (9)
C1	0.0812 (10)	0.0904 (12)	0.0836 (10)	−0.0030 (9)	0.0229 (8)	−0.0078 (9)
C2	0.1233 (17)	0.1070 (17)	0.1222 (18)	0.0007 (14)	−0.0045 (15)	−0.0236 (14)
C3	0.1254 (19)	0.138 (2)	0.125 (2)	0.0128 (18)	−0.0198 (16)	−0.0143 (18)
C4	0.1270 (18)	0.141 (2)	0.1022 (16)	0.0355 (17)	0.0072 (14)	0.0023 (16)
C5	0.176 (3)	0.1129 (19)	0.124 (2)	0.0376 (19)	−0.002 (2)	−0.0146 (16)
C6	0.1273 (17)	0.1016 (16)	0.1074 (15)	0.0120 (14)	−0.0010 (14)	−0.0146 (13)
N1	0.1038 (11)	0.0985 (12)	0.0866 (10)	0.0089 (9)	0.0158 (9)	0.0029 (8)
C7	0.0883 (11)	0.0872 (12)	0.0932 (11)	−0.0069 (9)	0.0257 (9)	−0.0135 (9)
C8	0.0905 (11)	0.0770 (10)	0.0880 (11)	−0.0035 (8)	0.0238 (9)	−0.0064 (8)
C9	0.0930 (11)	0.0749 (10)	0.0916 (11)	−0.0034 (8)	0.0275 (9)	−0.0068 (9)
C10	0.0902 (10)	0.0682 (9)	0.0866 (10)	−0.0003 (8)	0.0277 (9)	0.0002 (8)
C11	0.0892 (10)	0.0692 (9)	0.0913 (11)	0.0045 (8)	0.0253 (9)	0.0009 (8)
C12	0.0975 (11)	0.0703 (10)	0.0900 (11)	0.0007 (8)	0.0275 (9)	−0.0055 (8)
C13	0.0908 (10)	0.0794 (10)	0.0786 (10)	0.0002 (8)	0.0241 (8)	0.0067 (8)
C14	0.1093 (13)	0.0786 (11)	0.0981 (13)	0.0192 (10)	0.0211 (11)	0.0053 (10)
C15	0.1172 (14)	0.0709 (10)	0.0957 (12)	0.0115 (10)	0.0205 (11)	−0.0066 (9)
C16	0.1193 (17)	0.138 (2)	0.1126 (17)	0.0095 (16)	0.0036 (14)	−0.0324 (16)
C17	0.1095 (15)	0.1255 (19)	0.1013 (14)	0.0171 (14)	0.0150 (12)	0.0159 (13)

### Geometric parameters (Å, °)

**Table:**

F1—C4	1.349 (3)	C9—C10	1.434 (3)
O1—C7	1.231 (2)	C9—H9A	0.9300
C1—C2	1.374 (3)	C10—C15	1.386 (3)
C1—C6	1.381 (3)	C10—C11	1.403 (2)
C1—C7	1.481 (3)	C11—C12	1.366 (3)
C2—C3	1.366 (4)	C11—H11A	0.9300
C2—H2A	0.9300	C12—C13	1.407 (3)
C3—C4	1.330 (4)	C12—H12A	0.9300
C3—H3A	0.9300	C13—C14	1.403 (3)
C4—C5	1.368 (4)	C14—C15	1.368 (3)
C5—C6	1.371 (4)	C14—H14A	0.9300
C5—H5A	0.9300	C15—H15A	0.9300
C6—H6A	0.9300	C16—H16A	0.9600
N1—C13	1.356 (3)	C16—H16B	0.9600
N1—C16	1.440 (3)	C16—H16C	0.9600
N1—C17	1.454 (3)	C17—H17A	0.9600
C7—C8	1.460 (3)	C17—H17B	0.9600
C8—C9	1.333 (3)	C17—H17C	0.9600
C8—H8A	0.9300		
			
C2—C1—C6	117.0 (2)	C15—C10—C11	115.62 (17)
C2—C1—C7	119.2 (2)	C15—C10—C9	120.08 (17)
C6—C1—C7	123.72 (19)	C11—C10—C9	124.30 (17)
C3—C2—C1	122.6 (3)	C12—C11—C10	121.92 (17)
C3—C2—H2A	118.7	C12—C11—H11A	119.0
C1—C2—H2A	118.7	C10—C11—H11A	119.0
C4—C3—C2	118.0 (3)	C11—C12—C13	121.86 (17)
C4—C3—H3A	121.0	C11—C12—H12A	119.1
C2—C3—H3A	121.0	C13—C12—H12A	119.1
C3—C4—F1	118.5 (3)	N1—C13—C14	121.39 (17)
C3—C4—C5	123.0 (3)	N1—C13—C12	122.34 (18)
F1—C4—C5	118.4 (3)	C14—C13—C12	116.27 (17)
C4—C5—C6	118.0 (3)	C15—C14—C13	120.71 (18)
C4—C5—H5A	121.0	C15—C14—H14A	119.6
C6—C5—H5A	121.0	C13—C14—H14A	119.6
C5—C6—C1	121.3 (2)	C14—C15—C10	123.54 (18)
C5—C6—H6A	119.3	C14—C15—H15A	118.2
C1—C6—H6A	119.3	C10—C15—H15A	118.2
C13—N1—C16	121.72 (19)	N1—C16—H16A	109.5
C13—N1—C17	120.40 (18)	N1—C16—H16B	109.5
C16—N1—C17	117.76 (19)	H16A—C16—H16B	109.5
O1—C7—C8	121.0 (2)	N1—C16—H16C	109.5
O1—C7—C1	118.93 (18)	H16A—C16—H16C	109.5
C8—C7—C1	120.03 (17)	H16B—C16—H16C	109.5
C9—C8—C7	121.22 (18)	N1—C17—H17A	109.5
C9—C8—H8A	119.4	N1—C17—H17B	109.5
C7—C8—H8A	119.4	H17A—C17—H17B	109.5
C8—C9—C10	129.98 (18)	N1—C17—H17C	109.5
C8—C9—H9A	115.0	H17A—C17—H17C	109.5
C10—C9—H9A	115.0	H17B—C17—H17C	109.5
			
C6—C1—C2—C3	2.2 (4)	C8—C9—C10—C15	175.4 (2)
C7—C1—C2—C3	179.4 (3)	C8—C9—C10—C11	−4.1 (3)
C1—C2—C3—C4	−0.5 (5)	C15—C10—C11—C12	−1.3 (3)
C2—C3—C4—F1	−179.8 (3)	C9—C10—C11—C12	178.19 (17)
C2—C3—C4—C5	−2.5 (6)	C10—C11—C12—C13	−1.2 (3)
C3—C4—C5—C6	3.4 (5)	C16—N1—C13—C14	−179.2 (2)
F1—C4—C5—C6	−179.2 (3)	C17—N1—C13—C14	4.7 (3)
C4—C5—C6—C1	−1.5 (5)	C16—N1—C13—C12	0.7 (3)
C2—C1—C6—C5	−1.2 (4)	C17—N1—C13—C12	−175.38 (19)
C7—C1—C6—C5	−178.2 (2)	C11—C12—C13—N1	−176.80 (18)
C2—C1—C7—O1	−11.6 (3)	C11—C12—C13—C14	3.1 (3)
C6—C1—C7—O1	165.4 (2)	N1—C13—C14—C15	177.5 (2)
C2—C1—C7—C8	167.9 (2)	C12—C13—C14—C15	−2.4 (3)
C6—C1—C7—C8	−15.1 (3)	C13—C14—C15—C10	−0.1 (3)
O1—C7—C8—C9	−2.9 (3)	C11—C10—C15—C14	2.0 (3)
C1—C7—C8—C9	177.56 (17)	C9—C10—C15—C14	−177.53 (19)
C7—C8—C9—C10	−179.71 (19)		

### Hydrogen-bond geometry (Å, °)

**Table:**

*D*—H···*A*	*D*—H	H···*A*	*D*···*A*	*D*—H···*A*
C17—H17A···O1^i^	0.96	2.56	3.525 (3)	180

Symmetry codes: (i) −*x*+1, −*y*+1, −*z*+1.
